# Proteome-wide comparison between the amino acid composition of domains and linkers

**DOI:** 10.1186/s13104-018-3221-0

**Published:** 2018-02-09

**Authors:** Daniel Brüne, Miguel A. Andrade-Navarro, Pablo Mier

**Affiliations:** 1 Institute of Pharmacy and Molecular Biotechnology, Ruprecht Karls University Heidelberg, 69120 Heidelberg, Germany; 2 Faculty of Biology, Johannes Gutenberg University Mainz, Gresemundweg 2, 55128 Mainz, Germany

**Keywords:** Amino acid composition, Domains, Linkers

## Abstract

**Objective:**

Amino acid composition is a sequence feature that has been extensively used to characterize proteomes of many species and protein families. Yet the analysis of amino acid composition of protein domains and the linkers connecting them has received less attention. Here, we perform both a comprehensive full-proteome amino acid composition analysis and a similar analysis focusing on domains and linkers, to uncover domain- or linker-specific differential amino acid usage patterns.

**Results:**

The amino acid composition in the 38 proteomes studied showcase the greater variability found in archaea and bacteria species compared to eukaryotes. When focusing on domains and linkers, we describe the preferential use of polar residues in linkers and hydrophobic residues in domains. To let any user perform this analysis on a given domain (or set of them), we developed a dedicated R script called RACCOON, which can be easily used and can provide interesting insights into the compositional differences between a domain and its surrounding linkers.

**Electronic supplementary material:**

The online version of this article (10.1186/s13104-018-3221-0) contains supplementary material, which is available to authorized users.

## Introduction

Amino acid composition has been used in several studies to deduce properties of proteins, protein families and proteomes [1–3]. Amino acids are not randomly used in proteins but selected in evolution for their chemical properties in a sequence specific context. Importantly, part of this context is structural. Amino acid composition is strongly influenced by the exposure of the residues, which differs between the surface and the core of protein structures [4, 5]. Globular domains have therefore different constraints in their amino acid composition than linkers. However, there are no studies comparing amino acid composition of domains and linkers. To address this issue, we studied how amino acid composition in domains differs to the one of the linkers connecting them. Depending on the functionality given by a domain, its associated linker would require a certain amino acid sequence to provide a suitable environment for it, as linkers play a role in the regulation of the domain functions [6]. We considered 38 proteomes to characterize the differences between archaea, bacteria and eukaryotes. Finally, we focused on the case of DNA-binding domains to showcase how the consideration of amino acid composition of domains and linkers can be used to gain insight into the relation between protein sequence and function. We illustrate this example with a dedicated R script we developed called RACCOON.

## Main text

### Methods

We selected 38 complete and well-annotated reference proteomes (Additional file 1). They were obtained from UniProt [7], release 2016_01. For the whole-proteome amino acid composition study, all sequences were considered; when studying domains and linkers, proteins without annotated domains were discarded. Linkers were defined as sequences flanked by two domains. A file containing all SMART domains with a description of the domain functions was downloaded from SMART [8]. We use this list as a dictionary of all possible domain names.

Plots of the results were created using the *ggplot2* [9] and *scales* [10] R packages. The R packages *dplyr* [11] and *reshape2* [12] were used for data handling.

### Results

**Fig. 1:**
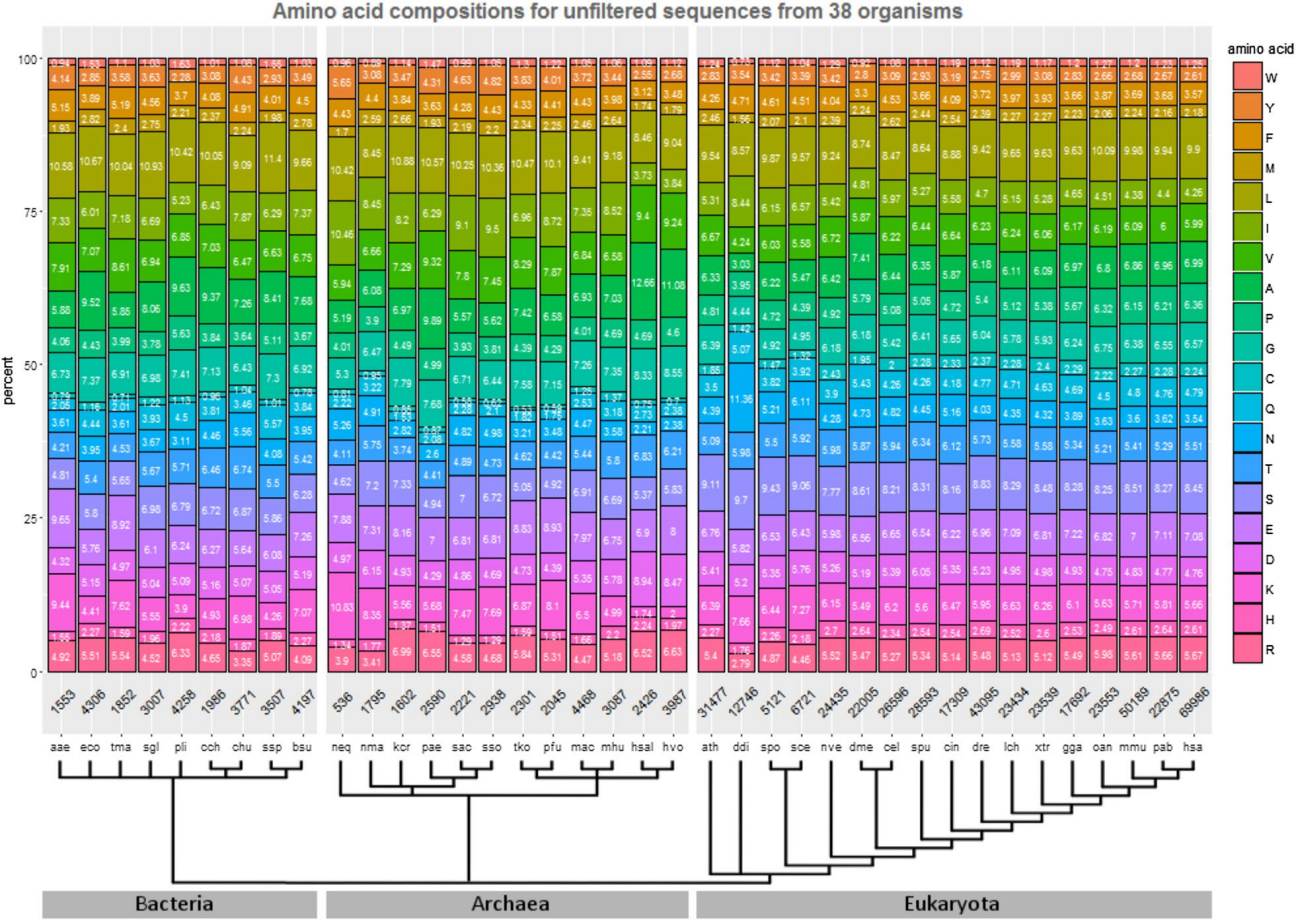
Amino acid composition of 38 reference proteomes. The phylogenetic relationship between the species can be seen in the tree beneath the species’ name abbreviations. The number of protein sequences extracted from each proteome is shown at the base of the bars

The proteomes of 38 species were first analyzed with respect to their proteome-wide amino acid composition. The observed differences are larger in archaea than in bacteria, and in bacteria than in eukaryotes (Fig. 1). Eukaryotes have the highest variability for proline, cysteine and asparagine. Amino acids that in general show high variability across species are lysine, alanine and isoleucine, while histidine, tryptophan and methionine vary the least. Cysteine is more common in eukaryotes than in archaea and bacteria, while isoleucine is less abundant in eukaryotes. *Dictyostelium discoideum* (ddi) stands out given its high proportion of asparagine, glutamine and isoleucine, and low proportion of alanine, valine and arginine [13]. The genome of *D. discoideum* is A+T-rich, thus the high proportion in N, Q and I, which are encoded in codons with high A+T content, while the amino acids with decreased frequencies are encoded by codons with higher G+C content.

**Fig. 2:**
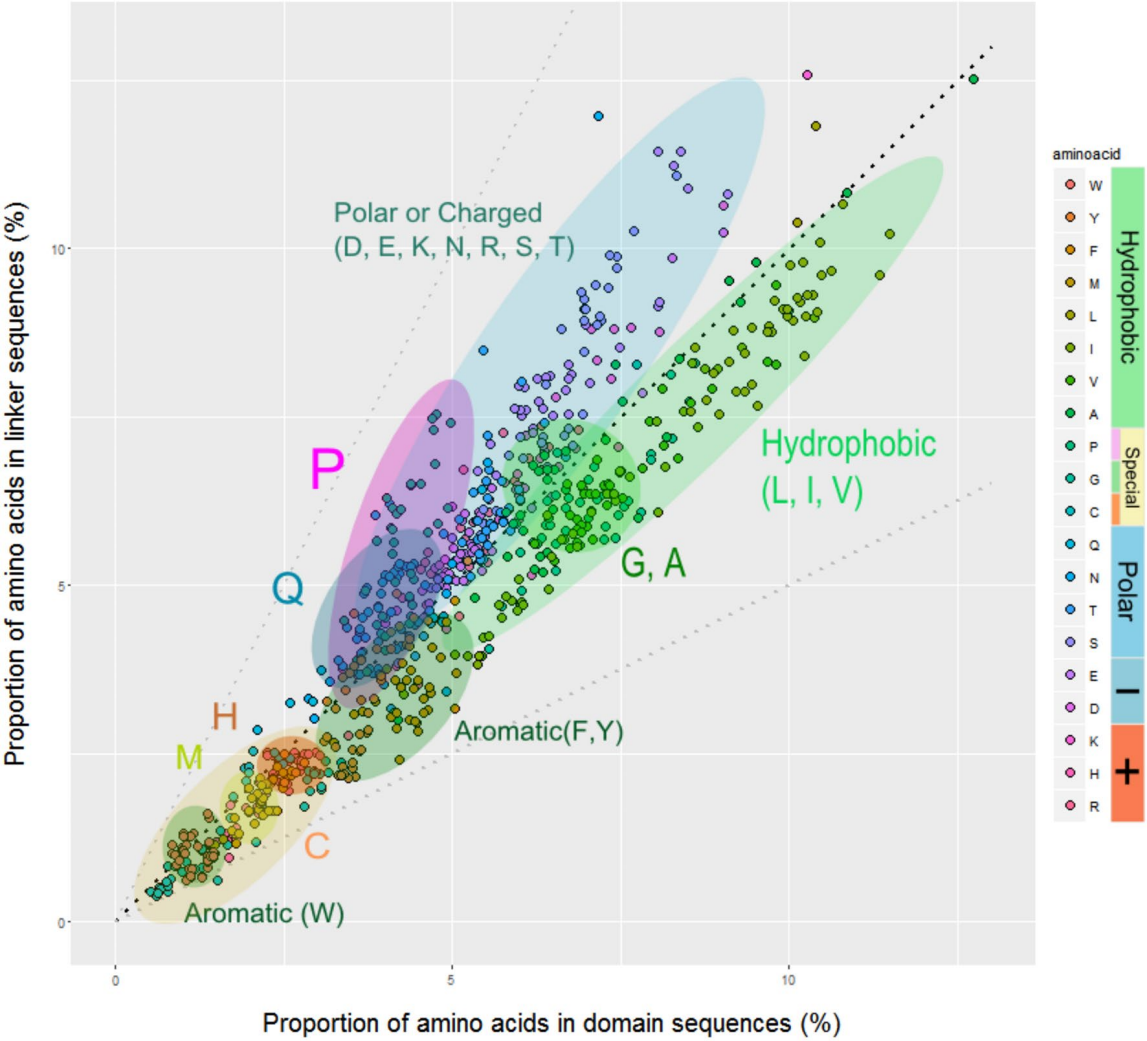
Differential amino acid usage in domains vs. linkers in each proteome. Each dot represents the percentage of use of an amino acid in one of the proteomes in linkers versus domains. The black dashed line is the bisect, while the grey dashed lines mark the twofold increase, so that the amino acids on these lines are either twice as abundant in linkers than in domains (upper dashed line) or twice as abundant in domains (lower dashed line)

Next, we studied the differential usage of amino acids in domains and linkers. For each of the species, we calculated their amino acid composition considering only regions annotated either as domains or linkers (Fig. 2). Proline and glutamine, but also less specifically, polar and charged amino acids, are more common in linkers. Amino acids more common in domains are the ones with hydrophobic side chains like leucine and valine, as well as the aromatic phenylalanine and tyrosine. These results were expected, since domains tend to be globular and linkers are more exposed, thus tend to have more polar or charged residues.

To allow any user to compare the amino acid composition of a specific annotated domain to the average amino acid composition in domains, in all species simultaneously (linkers accordingly), we developed an R script that uses the *shiny* framework [14]. It is called Relative Amino aCid Composition in dOmains and liNkers (RACCOON), and can be downloaded from our web site [15]. RACCOON allows the user to select a set of SMART domains by name or by string search of their names and descriptions. Once a set is selected, their amino acid composition is compared to that of the background of all domains (in 38 proteomes). The same analysis is presented for the linkers of the selected domains. This second analysis seeks to discover trends in amino acid composition that could uncover biases (and thus functionality) associated to the domains considered. This analysis is exploratory but relevant, given our current understanding of protein function, which has so far focused more in globular domains than in less ordered regions. The increasing evidence indicating that disordered regions have roles in regulation, interaction and disease, motivates this effort.

**Fig. 3:**
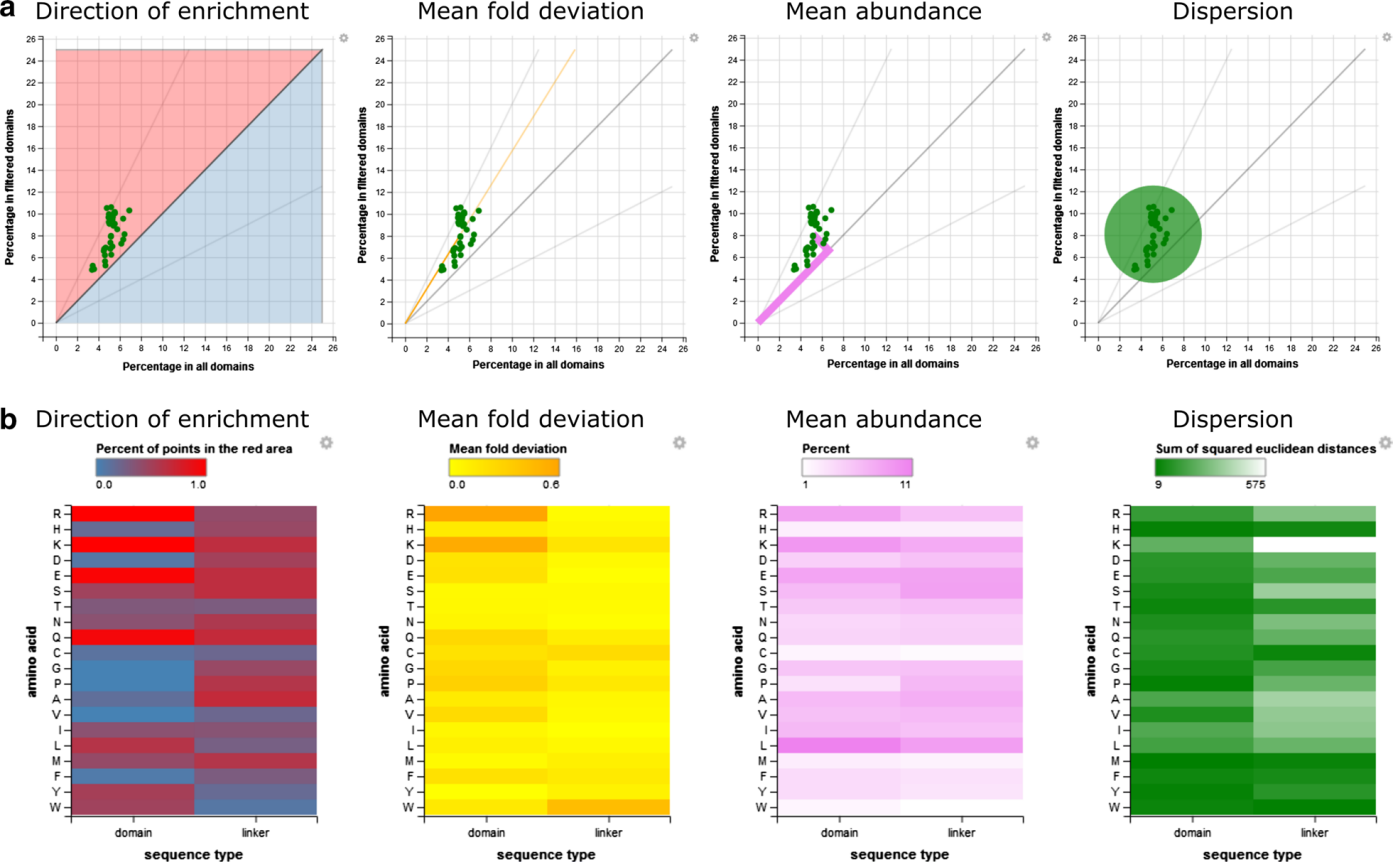
Composition of DNA-binding domains and linkers surrounding them, compared to the average in domains/linkers. **a** Results obtained in RACCOON when selecting the amino acid Arg in domains (see details in main text). **b** Direction of enrichment of average over-representation in the selected domains and surrounding linkers versus all domains and linkers in all the proteomes, mean fold deviation of amino acid usage over all proteomes, average percentage of amino acid usage over all the proteomes, and dispersion of the values (sum of squared Euclidean distances to centroid)

To illustrate our approach, we selected a set of domain names using in RACCOON the regular expression “DNA-binding|DNA binding” and including domains from SMART whose description matches the query (Fig. 3). The properties of each amino acid are compared between the desired feature (selected domains or their surrounding linkers) and the corresponding background (all domains or all linkers, respectively). Figure 3a illustrates the results for Arg in DNA-binding domains (green dots). Then, different variables are computed to represent the distribution of these values.

Two variables compare the fraction of each amino acid in the domain or linkers selected versus all domains or linkers: direction of enrichment and mean fold deviation. Direction of enrichment is the fraction of the proteomes for which a given amino acid is more present than in the background. A value of 1 indicates that in all proteomes considered the given amino acid was more frequent in the feature. Mean fold deviation is |(f/b) − 1|, where f is the mean percentage of the amino acid in the selected feature and b the mean percentage of the amino acid in the background; higher values indicate that the distribution deviates from the background. The direction is given by the direction of enrichment previously calculated.

Mean abundance is a variable that describes the amino acid usage percentage just in the selected feature without contrast to the background. Finally, dispersion is the sum of squared Euclidean distances of the proteomes to the average point of their distribution; large values indicate higher variability between species.

When we compute the values for all the residues (Fig. 3b), we can see the values we obtained for Arg in context: Arg usage in DNA-binding domains is consistently higher in all proteomes than in the background (direction of enrichment = 1) as it is the case for Lys, Gln and Glu. The separation of Arg in DNA-binding domains from the background is large (mean fold deviation = 0.6), only comparable to that of Lys. Its mean abundance makes Arg one of the most frequent residues in DNA-binding domains, comparable to Leu, which was not enriched. Arg usage in DNA-binding domains is more variable over the proteomes (5–11%) than in the background of all domains (3–7%), resulting in an average value for dispersion.

The results for the linkers surrounding DNA-binding domains are very different: they show Arg and Lys percentages similar to the background of linkers. Both linkers and domains are enriched in Gln, and linkers are enriched in Ser while domains are not. Serines in disordered regions are often target of phosphorylation [16], and could indicate that linkers surrounding DNA-binding domains hold many potential regulatory sites. The high percentage of Gln could be due to polyQ stretches, which are more abundant in proteins with many interaction partners, a property of nuclear proteins and, particularly, of transcription factors [17], which are DNA-binding proteins. Both Ser and Glu show high dispersion in the linkers and not in the domains, suggesting that this property might change among the species considered.

Nuclear proteins are known to have high levels of Arg and Lys; this could be due to arginine/lysine-rich motifs that are used as nuclear localization signals, which have been described to overlap or be adjacent to DNA-binding domains [18]. An additional explanation is that they are used to interact with the negatively charged DNA sugar-phosphate backbone [19]. The fact that this enrichment is not shown in linkers hints at a function that requires a structured region, thus indicating that specificity in the recognition of the DNA (or protein) partner is the general mechanism required.

### Discussion

The amino acid composition analysis of the proteomes reveals high heterogeneity between species, especially among archaea and bacteria (Fig. 1). This might be because they are highly heterogeneous both in their genomic architectures and in their environments. The amino acid composition in eukaryotes is less heterogeneous, particularly within multicellular species, except for *D. discoideum* with its Q and N-rich proteome [20].

The differential use of amino acids in domains and linkers (Fig. 2) illustrates a pattern of over-represented hydrophilic amino acids in linkers and hydrophobic amino acids in domains. An important fraction of protein folding energy is provided by the hiding of hydrophobic surfaces in the protein interior [21], thus the preferential use of hydrophobic amino acids in the well-structured domain regions. Conversely, the more flexible linker regions require a higher solubility, which explains the over-representation of hydrophilic amino acids in these regions. One exception is proline, which possesses a hydrophobic side chain and would be expected to be less used in linkers. Proline does not allow alpha-helices to continue and induces disorder in the surrounding protein structure, due to its special structure [22]. Thus, it is well suited to induce the transition from a well-structured domain to a more flexible linker. The amino acids showing almost no over-representation in any case are generally low in abundance and contain special functional groups, like cysteine and methionine, which contain sulfur, or histidine and tryptophan, which harbor nitrogen-containing aromatic rings.

The amino acid composition found in specific domains and their surrounding linkers provides the opportunity to analyze groups of domains with common characteristics or functions, and to check whether a certain amino acid profile can be extracted from them. For example, our analysis of DNA-binding domains suggests enrichments in amino acids in the linkers surrounding these domains that could be indicative of functionality in these likely disordered regions. Additionally, this analysis points to features specific to the domain, thus suggesting that known biases in positively charged residues (Arg, Lys) might have functions related to structured parts of DNA-binding proteins. This exemplary analysis took into account a large number of domains with a common function. A caveat is that if the selection of domains is small, for example relative to a single domain with few examples, there might be skews in the results simply because one will be looking at a few protein families. To warn the user, in the plot showing the values for an amino acid in RACCOON (Fig. 3a), the dots are colored depending on the number of domains or linkers matched by the query: green if more than ten; red if less than ten but more than five; and yellow otherwise.

In conclusion, we introduced the analysis of amino acid composition, distinguishing domains and their linkers, as a valuable tool to assess another layer of information from protein sequences. The combined analysis of domains and linkers provides interesting insights into their compositional differences and can give further pieces of evidence for models of molecular interactions and for the prediction of protein function.

## Limitations

- The present research is limited to 38 proteomes. It could be further extended to include a greater number of completely sequenced species.
- As we depend on the domain annotation given by UniProt, domains not yet annotated in a sequence are lost in our analysis, as we do not consider unannotated regions.
- The conclusions drawn from the amino acid composition of a specific set of domains or linkers may be due to the skewed representation of these domains in the database.
- To use RACCOON, the user needs some previous bioinformatic knowledge. In our web site we have included a detailed “How to” section with easy steps to simplify its use.

## Additional file

**Additional file 1.** List of proteomes used for the analyses. Each proteome is described by the name of the species, abbreviation as used in the manuscript, UniProt organism ID, number of proteins, and percentage of amino acids from domains/linkers against the total amino acid composition of the proteome.

## Abbreviations

RACCOON: Relative Amino aCid Composition in dOmains and liNkers
ddi: *Dictyostelium discoideum*
